# Recurrence hernia in the broad ligament of the uterus: a case report

**DOI:** 10.1186/s40792-020-01030-5

**Published:** 2020-11-16

**Authors:** Yoshifumi Hashimoto, Tatsuo Kanda, Tadasu Chida, Kazuyoshi Suda

**Affiliations:** 1 Department of Surgery, Sanjo General Hospital, TsukanomeNiigata, Sanjo 955-0055 Japan; 2 Department of Surgery, Saiseikai Sanjo Hospital, Sanjo, Japan

**Keywords:** Broad ligament of uterus, Internal hernia, Recurrence

## Abstract

**Background:**

Bowel herniation through a defect in the broad ligament of the uterus is a rare disease and few cases of recurrence have been reported. We report herein a recurrence case of a patient with broad ligament hernia (BLH), along with a review of the literature.

**Case presentation:**

A 53-year-old woman complaining of abdominal pain was transported to our hospital. She had a history of laparotomy for small-bowel obstruction associated with hernia in the broad ligament of the uterus 10 years ago at a local hospital. Abdominal pelvic contrast-enhanced computed tomography revealed that the mesentery of the dilated bowels converged at a thick band in the pelvis, suggesting closed loop obstruction of the small bowel. The patient underwent urgent laparotomy and was diagnosed with bowel herniation through an opening in the broad ligament of the uterus on the right side, which was ipsilateral with the previous surgery. The hernia orifice was widened by incision and incarcerated bowel segments were released and preserved because ischemia was reversible. The membranous defect of BLH was closed by suture with braded silk strings.

**Conclusions:**

Although BLH is a rare disease, patients face a significant risk of disease recurrence. Nonabsorbable suture may be advisable for closure of the hernia orifice in BLH.

## Background

Bowel herniation through a defect in the broad ligament of the uterus is a rare disease. Defects in the broad ligament have been noted in only 0.5% of autopsy cases and the clinical presentation of broad ligament hernia (BLH) accounts for 4% to 5% of all internal herniations [1]. Owing to the rarity of BLH itself, its recurrence is even rarer. As to recurrence cases, there is no English literature and only one case reported in Japanese [2] is available. We recently experienced a recurrence case of BLH in a middle-aged woman and report that case herein because the present case has clinical implications in diagnosis, management, and surgery.

## Case presentation

A 53-year-old woman complaining of sudden onset of abdominal pain was transported to our hospital by ambulance at midnight. She had a history of two normal vaginal deliveries and small-bowel obstruction associated with BLH 10 years ago. In the latter, the patient underwent urgent surgery and ischemic bowel was excised in a local hospital.

**Fig. 1:**
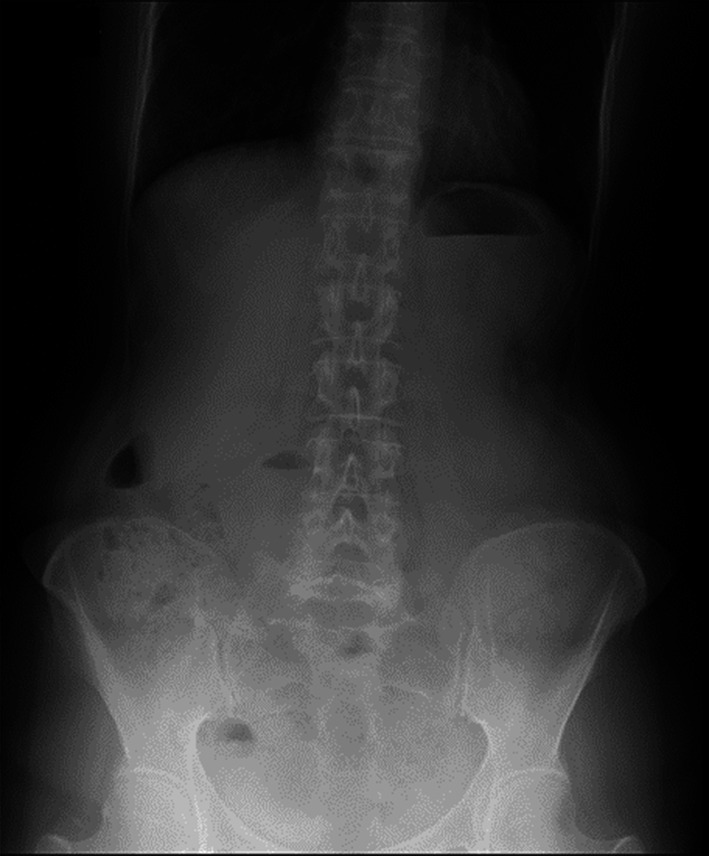
Abdominal radiographs on admission showed no evidence of bowel obstruction

Abdominal X-ray examination revealed no niveau formation (Fig. 1) although the patient mentioned that the symptoms were similar to those observed at the time of the previous surgery. There were no signs of bowel obstruction or peritonitis in physical examination. Hematological and biochemical examinations also yielded no abnormal findings. The patient was hospitalized and carefully observed overnight as an acute abdomen patient.

**Fig. 2:**
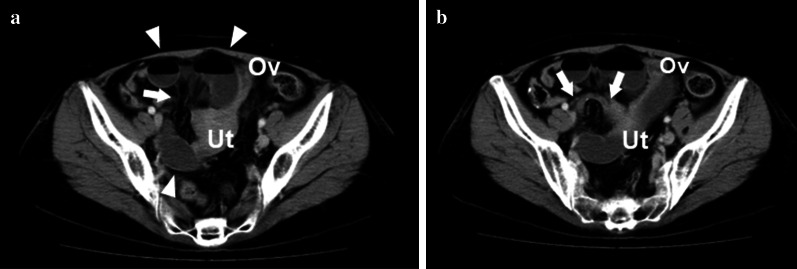
Contrast-enhanced computed tomography (CT) findings. Small-bowel loops (arrowheads) were dilated in the vicinity of the uterus and the mesentery converged at the right side of the uterus (arrow). The uterine showed counterclockwise rotation (**a**). The next slice of A revealed a thick band (arrows) interpolated in the convergence of the mesentery (**b**). Ut and Ov indicate uterine and left ovary, respectively

Because of no relief of abdominal pain, the patient underwent contrast-enhanced computed tomography (CT) the next morning. Abdominal CT showed that the mesentery of the dilated small bowels converged at the right side of the uterus where an intervening band was found (Fig. 2). From these findings, we decided urgent surgery because of closed-loop obstruction associated with bowel herniation in the right broad ligament of the uterus.

Laparotomy revealed that the ileum entered a small opening in the right broad ligament of the uterus, by which the ileal loop approximately 70 cm in length was strangulated. The right broad ligament was partially incised and the hernia orifice was widened to release the strangulated ileal loop. The incarcerated ileum was preserved because ischemia was reversible.

**Fig. 3:**
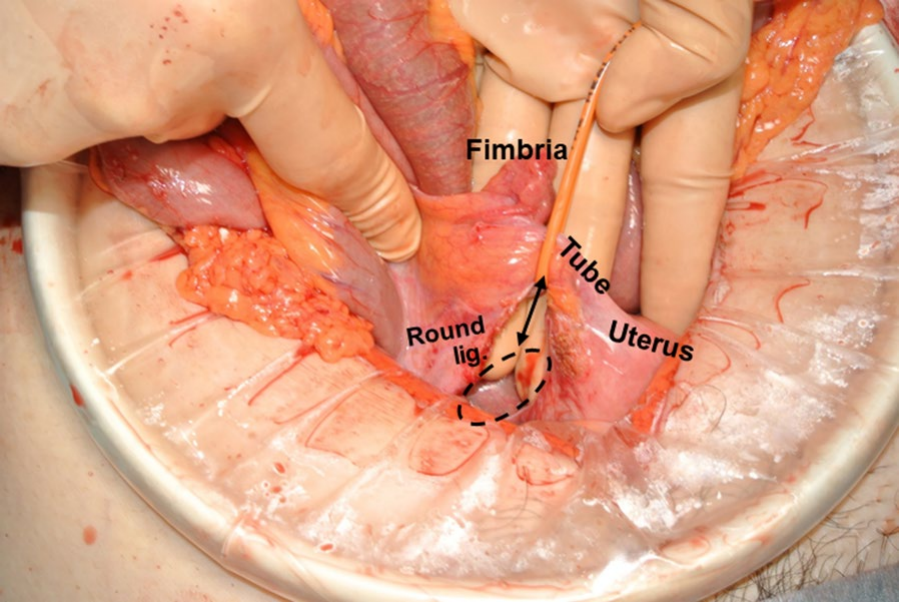
Surgical findings. Incarcerated ileal loop was released after widening the broad ligament of the uterus (double-headed arrow). The original hernia orifice (defect in the mesometrium) is depicted with a dotted oblong

The opening in the broad ligament measured 2.5 cm in diameter. The shape was oval and the margin was smooth (Fig. 3). To reinforce the mesometrium, the defect was repaired with “vest over pants” type sutures [3] using 3-0 silk strings.

The patient had a favorable postoperative course and was discharged on postoperative day 9. We obtained the surgical records from the hospital where the patient underwent the previous surgery for bowel obstruction 10 years ago. The bowel obstruction was caused by BLH on the right side, the same side as the present episode. The records showed that the defect in the broad ligament was closed with simple suture of the mesometrium although it did not mention the type of strings used.

## Discussion

BLH is a rare female-specific internal hernia that accounts for 4% to 5% of all internal hernias [1]. There has been an increase in the number of BLH cases reported in Japan [2, 4–7]. This trend may be associated with the advanced aging of the Japanese population, or could be explained by publication bias because most of the reports from Japan were related to laparoscopic surgery. Despite it being an intriguing issue, it remains unclear whether racial difference exists or not because precise data on the incidence of BLH are unavailable as yet.

To know whether recurrence cases of BLH have been reported or not, we searched the literature using PubMed database, selecting “broad ligament,” “hernia,” and “recurrence” as key words. Although we were able to get two hits, careful examination revealed that neither reported recurrence cases. Then, we searched the Japana Centra Revuo Medicina database in a similar manner and found one report of a recurrence case of BLH by Aisu et al. [2] in Japanese. In that case, a 49-year-old woman who had a history of left BLH two years before underwent laparoscopic surgery for the repair of ipsilateral BLH. They reported that the defect in the broad ligament was closed using absorbable suture in the first surgery.

**Fig. 4:**
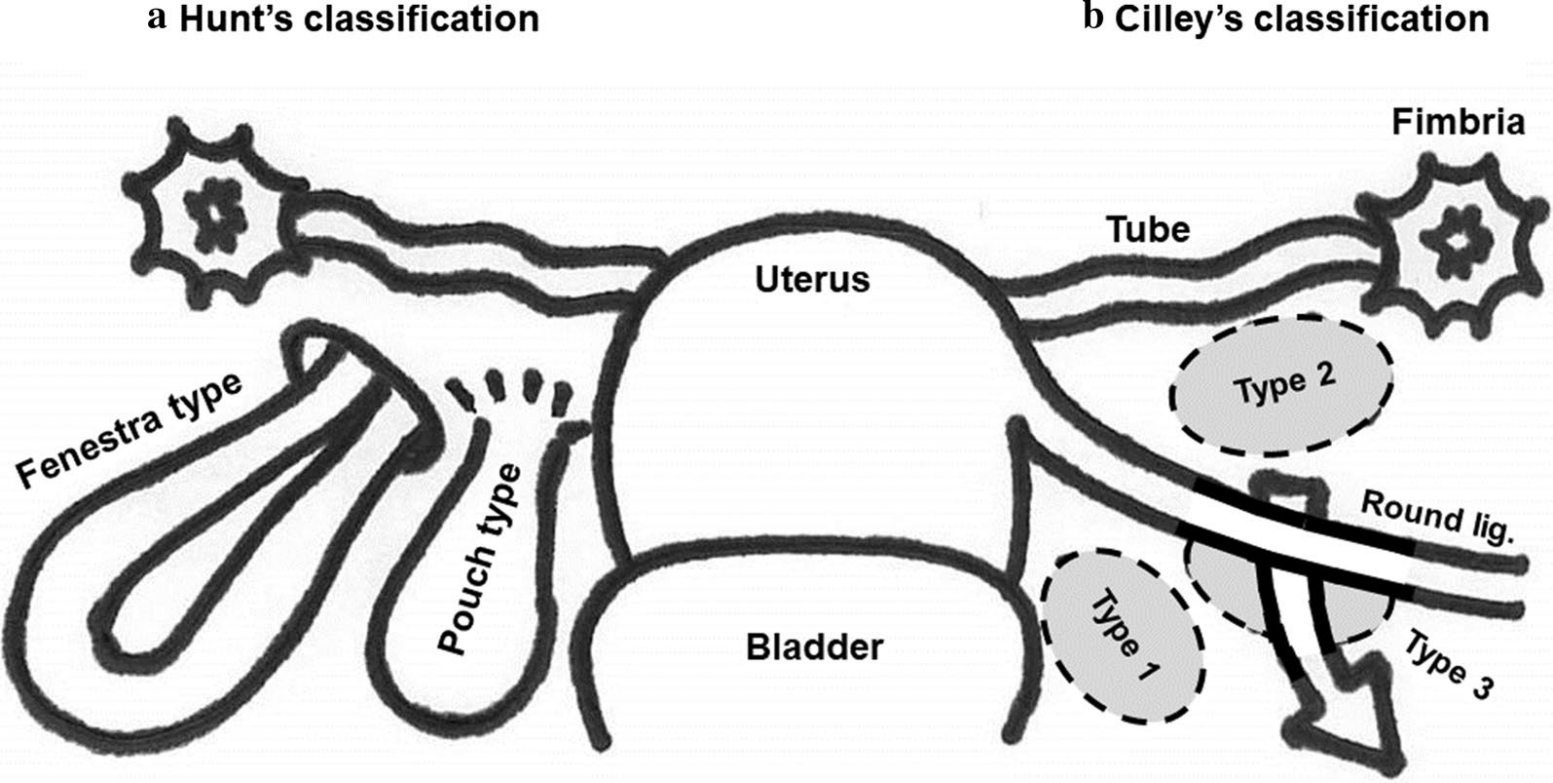
Two classifications of broad ligament hernia. Hunt’s classification divides the disease into fenestra and pouch types on the basis of the nature of the hernia defects (**a**). Cilley’s classification is anatomically based: in type 1, the defect develops caudal to the round ligament of the uterus; in type 2, the defect is located above the round ligament between the mesovarium and the mesosalpinx; and in type 3, the defect occurs in the mesoligamentum teres of the uterus (**b**). Dotted ovals represent openings and an arrow indicates the route of herniation in type 3

Two classifications are used for diagnosing BLH on the basis of the nature and the location of the defect. Hunt classified BLH into two types: fenestra and pouch, based on the nature of the defect [8]. The most common fenestra type is characterized by a complete two-layer defect of the broad ligament. The pouch type is a hernia forming a pouch caused by a one-layer defect or an attenuation of the mesometrium. Cilley’s classification is anatomically based and includes three types [9]. In type 1, the defect develops caudal to the round ligament of the uterus. In type 2, the defect is located above the round ligament between the mesovarium and the mesosalpinx, and in type 3, the defect occurs in the mesoligamentum teres of the uterus. We summarize those two classifications in a figure (Fig. 4). Our present case was classified as fenestra type and type 1, respectively.

As regards the mechanisms of the defect formation, two hypotheses are raised: congenital and acquired. A defect in the broad ligament of the uterus was observed in 0.5% of autopsy cases [10]. Rose [11] reported BLH in a 16-year-old girl, which could be important evidence that defects in the broad ligament are congenital at least in some BLH cases. On the other hand, Slezak et al. [12] pointed out that more than 85% of BLH cases occurred in parous women, suggesting that the decreased elasticity of the broad ligament associated with pregnancy or injury at delivery is relevant to the defect formation. The patient in the present case had a history of two deliveries. Furthermore, the fact that recurrence cases exist may support the hypothesis that the defects of BLH are acquired.

Diagnosing BLH was difficult prior to the prevalence of CT. Routine examinations including abdominal X-ray and laboratory tests yielded no abnormal findings in the present case. Contrast-enhanced CT is considered essential for the diagnosis of BLH [13]. BLH has the following characteristics: (1) a dilated small-bowel loop in the vicinity of the pouch of Douglas or uterus; (2) a shifted or compressed uterus, sigmoid colon, or rectum as a result of the small-bowel loop; and (3) congested mesentery converging at the broad ligament, which is associated with the small-bowel loop [14]. All these characteristics were noted in the present case, which prompted our decision for early intervention and resulted in successful bowel rescue. BLH has a high risk of strangulation and requires surgery for the reduction of herniated bowels. Clinicians should be aware of the above-mentioned characteristics when reading CT images of patients with acute abdomen, particularly in middle-aged parous patients.

Because of the rarity of BLH, a standard procedure for BLH repair has yet to be determined. As defects in the mesometrium are small in size, simple closure is generally selected [4–7, 13–15]. From their experience of the recurrence case, Aisu et al. [2]*.* proposed the use of non-absorbable strings for closure of a defect in the broad ligaments or a wide opening with incision of the fallopian tube and the round ligament as a more secure alternative than simple closure because BLH patients are presumed to have congenital weak mesometrium. Despite the lack of description of the strings used, we surmised that absorbable suture was used in the first surgery for BLH, given that the hospital routinely selected absorbable suture, and two-layer closure with non-absorbable suture was used to repair the mesometrial defect.

## Conclusions

We presented herein a patient with recurrence of BLH. In the present case, contrast-enhanced CT was helpful for treatment decision-making for early intervention, leading to the salvage of herniated bowels. Despite the low incidence, recurrence can happen in BLH as well as other types of hernia. Although there is no clinical evidence of surgical procedures for BLH repair, surgeons may have to devise repair at surgery, including the use of non-absorbable suture, two-layer closure, or wide opening with incision of the round ligament and the fallopian tube, to prevent recurrence.

## Data Availability

Data supporting the conclusions are included in the article.

## Abbreviations

BLH: Broad ligament hernia
CT: Computed tomography
